# Videoconferencing in mental health services for children and adolescents receiving child welfare services: a scoping review

**DOI:** 10.1186/s12913-024-11157-y

**Published:** 2024-06-14

**Authors:** Marian Ådnanes, Jannike Kaasbøll, Silje L. Kaspersen, Vibeke Krane

**Affiliations:** 1 Department of Health Research, SINTEF Digital, Trondheim, Norway; 2 Department of Mental Health, Norwegian University of Science and Technology (NTNU), Regional Centre for Child and Youth Mental Health and Child Welfare (RKBU Central Norway), Trondheim, Norway; 3 Department of Health, Social and Welfare Studies, Faculty of Health and Social Sciences, University of South-Eastern Norway, Drammen, Norway

**Keywords:** Child welfare service, Child and adolescent mental health services, Video consultation, Treatment, Interdisciplinary meetings

## Abstract

**Background:**

Videoconferencing is considered an alternative to face-to-face consultations and a possibility to help overcome access-to-care barriers in mental health care services. Barriers to child and adolescent mental health services are particularly apparent in the case of children and adolescents receiving child welfare services. This scoping review aims to provide an overview of research on videoconferencing in the mental health treatment of children and adolescents receiving support from child welfare services.

**Methods:**

This scoping review follows the review framework outlined by the Joanna Briggs Institute. The following databases were searched from January 2012 to April 2024: Scopus, Web of Science, PubMed, PsycINFO (Ovid), CINAHL Plus, Social Services Abstracts (ProQuest), Sociological Abstracts (ProQuest), and Google Scholar.

**Results:**

The search yielded 4322 unique records and resulted in the inclusion of 22 articles that met the inclusion criteria. The studies originated from Denmark, England, Australia, Norway, Canada, Chile, and the USA, and were grouped into four areas: (1) videoconferencing to increase access to mental health treatment for vulnerable groups (2) young people’s perspectives (3) videoconferencing in interdisciplinary collaborative meetings, and (4) use, awareness, and acceptance of videoconferencing among health and social care providers.

**Conclusions:**

This scoping review shows that if videoconferencing in mental health care is to become an established and trusted method aimed at children and adolescents receiving child welfare services, several unresolved and potentially negative issues need attention and more research. This particularly applies to whether videoconferencing decreases or exacerbates inequalities in access to mental health services. A further question is whether new barriers are raised by screen-based treatment to threaten good therapeutic relationships, and by extension treatment quality and clinical outcomes.

**Supplementary Information:**

The online version contains supplementary material available at 10.1186/s12913-024-11157-y.

## Introduction

Videoconferencing in mental health care services has been used for many years and has historically been a tool to improve access to treatment in rural areas [1]). Videoconferencing technology (e.g., computers, laptops, and smartphones) enables auditory and visual communication in real-time across great distances. Previous reviews on videoconferencing in child and adolescent mental health services (CAMHS) have concluded that videoconferencing can improve access to treatment and increase empowerment and satisfaction for the service users [2–6]. Disadvantages summarized in this literature include the difficulty of capturing non-verbal communication, the difficulty of achieving a good therapeutic relationship, and new barriers because of technological, legal, ethical, and administrative issues. Although these barriers to treatment can become particularly apparent in the case of vulnerable and underserved groups, for example children and adolescents in child welfare services (CWS), we have not identified reviews of literature that focus on this aspect. Child welfare services’ purpose is to help children and families in difficult situations aiming to ensure children’s safety and well-being. CWS varies significantly across countries, reflecting different legal, cultural, and social frameworks [7].

Children and adolescents receiving support from CWS have a high prevalence of psychiatric diagnoses and mental health challenges, yet receive poor mental health care in relation to their needs and compared to other children with the same challenges [8–11]. Several factors at individual and service levels contribute to the disparity between needs and provision of mental health services. A recent review show that young people (12–18 years old) receiving help from CWS or their carers may not engage in help seeking because of their concerns about stigmatisation, confidentiality, service inaccessibility, perceived lack of understanding of young people’s circumstances and few opportunities for participation in decision making [12]. They also experience a lack of continuity of care within CAMH-services due to frequent changes in therapists or social workers, which can hinder the development of trust and effective therapeutic relationships [13]. Moreover, in cases of more serious mental illness, adolescents tend to turn to their parents or teachers for help [14–16]. However, research indicates that parents of children receiving CWS are among those in least frequent contact with mental health services for their children’s mental health problems and disorders [17]. Another barrier for treatment is that these children have often experienced multiple losses in important relationships with child welfare professionals, which may make them more vulnerable to becoming involved in professional therapeutic relationships [18]. Challenges at service level are related to interagency collaboration between CWS and CAMHS [19, 20].

Videoconferencing increased significantly in health and social care during the COVID-19 pandemic [21, 22], due to the urgent need for alternatives to face-to-face consultations and interdisciplinary meetings. While therapists using videoconferencing during COVID-19 found their way through trial and error and with varying degrees of support and guidance [23], it is uncertain to what extent they will continue to use videoconferencing or whether they and their clients will prefer to go back to face-to-face consultations. There is a need for systematic knowledge about the use and consequences of videoconferencing in mental health treatment for children and adolescents receiving help from CWS. The aim of this scoping review was to provide an overview of existing peer-reviewed empirical research on the use of videoconferencing in CAMHS treatment of children and adolescents receiving help from CWS, and in interdisciplinary collaborative meetings between CAMHS and CWS.

## Method

This scoping review was designed to summarize existing knowledge from original articles on videoconferencing in CAMHS, aimed at children and young people receiving help from CWS, and interdisciplinary video meetings between CAMHS and CWS. We examined the scope and characteristics of the research, summarized the main findings, and identified gaps in the literature, which can provide direction for future research.

A scoping review is a type of knowledge synthesis that follows a systematic approach to explore the knowledge base and identify knowledge gaps, summarize literature, clarify terms, or examine concepts [24]. This study used the method for scoping reviews outlined by the Joanna Briggs Institute [25]. The scoping review process consisted of five stages: 1) identifying the research questions, 2) identifying relevant studies, 3) study selection, 4) charting the data, 5) collating, summarizing, and reporting results. In the following sections, these stages are described in further detail.

The following research question was explored: What is known about the use of videoconferencing in CAMHS treatment of children and adolescents receiving help from CWS, and in interdisciplinary meetings between CAMHS and CWS?

### Identifying relevant studies (search strategy)

According to the recommendations for scoping reviews of the Joanna Briggs Institute, the search strategy was guided by key inclusion criteria based on the “population-concept-context” framework (Table 1). Two researchers (MÅ, SLK) developed the search strategy in collaboration with a librarian at the independent research institute SINTEF. The search was designed to identify records containing the following two concepts: (1) the use of videoconferencing in CAMHS for children and young people in contact with CWS, and (2) use of video meetings in collaboration between CWS and CAMHS. The search was limited to original empirical studies published in peer-reviewed journals between January 2012 and April 2024. Only articles written in English were included.

**Table 1:** Inclusion and exclusion criteria based on the “population–concept–context” framework

Criterion	Inclusion	Exclusion
Population	Children and adolescents receiving CWS, or vulnerable groups^a^ of children and adolescents with barriers to treatment in CAMHS	Children and adolescents who do not receive CWS or represent a vulnerable group of children and adolescents with barriers to treatment in CAMHS; children in CWS who are not being treated in CAMHS
Concept 1	Videoconferencing in mental health treatment	Other digital tools than videoconferencing in connection with mental health treatment
Concept 2	Videoconferencing in interaction and cooperation between CWS and CAMHS (and any other services involved)	Other digital tools than videoconferencing in interaction and cooperation between CWS and CAMHS (and any other services involved)
Context	Empirical primary studies	Articles without empirical evidence (discussion articles), reviews, master’s theses, doctoral theses, conference papers, books. The language was limited to British or American English or Scandinavian languages (Norwegian, Swedish, and Danish)

^a^Studies mentioning barriers to CAMH-treatment among children and adolescents who are vulnerable based on their background or circumstance, without mentioning contact with CWS but where children and adolescents share the same factors of vulnerability, for example socioeconomic deprivation, living in rural areas, belonging to a minority group or victims of trauma exposure

A structured search in international literature was conducted using the following databases: Scopus, Web of Science, PubMed, PsycINFO (Ovid), CINAHL Plus, Social Services Abstracts (ProQuest), Sociological Abstracts (ProQuest), and Google Scholar.

The following search string was used in Scopus, Web of Science, PubMed, PsycINFO and CINAHL: (“child welfare” or “child protect*” or “residential youth care” or “residential child care” or “residential care” or “out of home care” or “group home” or “institutional care” or “children’s home” or “foster home care” or “foster home” or “foster care” or “foster parent”) and (videoconferencing or “video consultation” or “digital communication” or “digital tools” or digital or online or telemedicine or telepsychiatry or telehealth or e-health or e-therapy).

For searches in Social Services Abstracts and Sociological Abstracts, the term “online” was removed from the search string above. The reason for this was that the term did not provide additional information to the other search terms, but on the contrary gave irrelevant hits that referred to e.g., the fact that an online survey had been conducted in the study.

For searches in Google Scholar, the search string was as follows (due to reduced space for letters and characters): “child welfare service” or “child protective service” or “residential child care” or “foster care” and “mental health services” and “videoconferencing” or “video consultation”.

### Study selection

The study reviewed published research articles with empirical findings (i.e., not review articles, gray literature, opinion, or theoretical pieces). The time frame was chosen to review developments in recent years, with an expectation of finding an increase in publications in connection with the services’ adaptation to COVID-19. The inclusion and exclusion criteria are described in Table 1.

To be included in our summary, a study had to be empirical research providing new knowledge and/or testing existing knowledge, and containing a description of the methods of data collection and analysis.

**Fig. 1:**
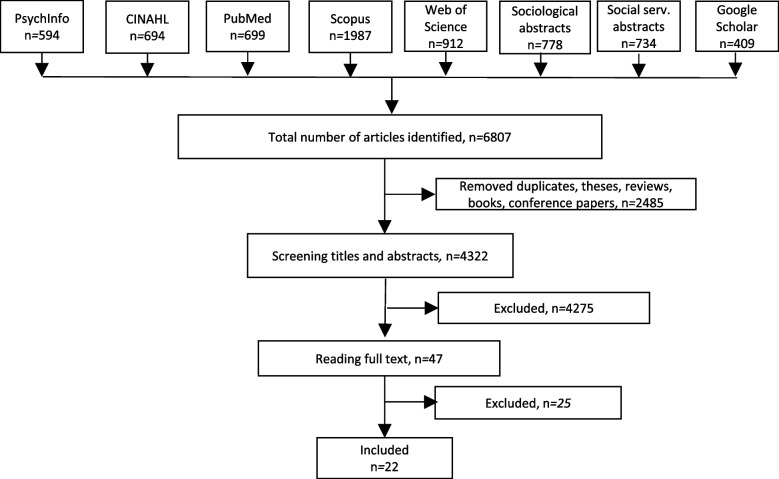
Flow chart of identified peer-reviewed empirical literature

Records from the different bibliographic databases were imported to EndNote X9 for Windows. Before manual screening commenced, duplicates were removed. Titles and abstracts of studies were screened for eligibility, based on the a priori inclusion criteria described above. The flowchart in Fig. 1 below shows the selection process resulting in the 22 included studies.

### Data extraction, collating, summarizing, and reporting the results

Titles and abstracts were independently screened by MÅ, SLK and VK (not double screened). Based on the methodology for scoping reviews of the Joanna Briggs Institute, data were extracted from the included studies using a data charting form (Excel file). The first author (MÅ) went through all papers included for full text reading, and extracted the following information: authors, title and year of publication, country (where the study was conducted), background for the study, aims, services and type of service users, element of videoconferencing in the study, method and data (i.e., qualitative or quantitative), findings/results related to the use of videoconferencing, and conclusion. The second author (SLK) reviewed the extracted data. Our coding system and categories facilitated the thematic analysis reported in the results section. Recurring themes in the primary findings identified throughout the coding process were progressively noted in the database. The summary of findings for each article was then re-read and coded based on the common themes.

## Results

The search provided 4322 unique records to be screened and resulted in the inclusion of 22 research articles that matched the criteria for the search. The 22 publications originated from Denmark (1), UK (6), Australia (1), Canada (2), Chile (1), Norway (1) and the USA (10). One study was mainly performed in England (UK), but also included interviews from Australia, Israel, New Zealand, Republic of Ireland and the USA [26]. In terms of methodology, 14 articles were qualitative, seven were quantitative and two used mixed methods. Three publications before the year 2020 were included: one from 2017, one from 2018 and one from 2019, while there were none from 2020, seven from 2021, four from 2022, seven from 2023 and one from 2024. Of 19 publications published after 2020, 15 focused on the impact of COVID-19 in terms of the introduction, experiences, and further use of videoconferencing/video consultations and digital meetings in this field.

Further presentation of the 22 studies is organized based on the four main themes identified in our analysis: (1) videoconferencing to increase access to CAMH treatment (for vulnerable groups) (2) young people’s perspectives (3) videoconferencing in interdisciplinary meetings, and (4) use, awareness, and acceptance of videoconferencing in CAMHS and CWS. The articles are presented in Table S-1 in Supplementary material.

## Theme 1: videoconferencing to increase or ensure access to CAMH treatment

Ten of the studies investigate the use of videoconferencing as a tool in different strategies to improve access to and completion of CAMH treatment for vulnerable and underserved children and young people receiving CWS, and related groups with barriers to treatment [27–36]. Two of these were published before COVID-19 [32, 36], during or after the pandemic, focusing on the increased relevance of technology based solutions for mental health care to ensure access, feasibility and continuity of care. Most of the studies within theme 1 had only service providers as informants – from health services, social services, schools or other agencies [27, 28, 30–32, 34]. Two of the studies were based on health records [33, 35] one of them also included focus groups with mental health providers [33]. Three of the studies are qualitative [28, 30, 31], five quantitative [27, 29, 32, 35, 36] and two uses mixed methods (qualitative and quantitative) [33, 34].

### Objectives and methods in studies under theme 1 (access to treatment):

Three of the studies explore teletherapy to children who have experienced trauma [27, 33, 36]. Two of them explore trauma-focused cognitive behavioral therapy (TF-CBT) delivered via telehealth technology [33, 36]. Stewart et al. [36] reported outcomes from before to after one-to-one video consultations in a pilot study (*n* = 15) directed at underserved, vulnerable children and adolescents, with at least one barrier to treatment (economically disadvantaged, living in rural areas, belonging to a racial or ethnic minority group). Data were collected using standardized self-report and caregiver report instruments to measure children’s symptoms and satisfaction with the service: The UCLA PTSD RI [37], Short Mood Feeling Questionnaire [38], Screen for Children’s Anxiety-Related Emotional Disorders [39], and Child and Parent Versions and Child Behavior Checklist [40]. Martin et al. [33] studied feasibility of the same therapy as Stewart et al. [36] (TF-CBT), delivered to young people in foster care in the United States at an integrated primary care clinic exclusively serving young people in care. Martin et al.’s [33] study was motivated by the high rate of trauma exposure in this population and at the same time systematic and patient barriers that inhibit treatment. They examined outcomes for patients who received telehealth (TF-CBT), along with factors that may have impacted successful completion through patient data collected retrospectively from the electronic health records of 46 patients who received telehealth TF-CBT between March 2020 and April 2022 [33].

Baker et al.’s [27] study on teletherapy, designed for traumatized children, was designed to ascertain challenges and opportunities presented by the widescale usage of teletherapy especially for traumatized children, necessitated by the COVID-19 pandemic and with data collected through an online survey among 250 clinicians across the United States, including five questions about the perceived positive impact of teletherapy on logistical aspects: safety, access, convenience, scheduling, and attendance [27].

The survey by Malas et al. [32] was motivated by the lack of access to mental health services in primary care, particularly pointing out the need for child and adolescent psychiatrists. The authors introduced and tested a telepsychiatry program offering behavioral health consultants in primary care, telephonic consultations, video consultations and embedded care. They assessed primary care providers, child and adolescent psychiatrists and pediatricians over a five-year period (*n* = 649), asking questions about their attitudes and perceptions regarding the telepsychiatry program including consultation, efficiency, user-friendliness, and confidence in providing mental health care [32].

Mundt and colleagues [34] evaluated the feasibility of a telepsychiatry consultation program for primary health care and treatment of institutionalized children and adolescents with complex mental health care needs, living under the supervision of CWS. Scarce and uneven distribution of specialized mental health teams in Chile and limited provision and quality of care for this vulnerable population were important background factors, and these inequities were addressed through telepsychiatry. The program consisted of 90-min mental health video consultations (*n* = 15) involving eleven children and eight clinicians, scheduled twice per month over a six-month period, delivered by child and adolescent psychiatrists based in Santiago, Chile. The study collected data about clinician-rated usefulness and acceptability, clinical patient information and actions taken or agreed on for patient management, in addition to the number and types of health care providers participating in each session, duration of sessions and technology used [34].

Poorer access to mental health care due to social inequality was the background for the study by Eapen et al. [28], aiming to extend the reach of and access to evidence-based, trauma-focused treatment, particularly for individuals disadvantaged by inequity during COVID-19. Eapen et al. [28] point out significant barriers based on economically poor background, living in rural areas, and belonging to a racial or ethnic minority group, despite the high prevalence of trauma exposure among these underserved groups. The authors performed a lexical analysis of clinicians’ reflections as they delivered psychiatry services to children and families in New South Wales (*n* = 6), aiming to explore the advantages and disadvantages of the transition to e-mental health from the perspective of service providers.

The aim of the study by Loria et al. [31] was to increase knowledge about the perceived impact of COVID-19 on the health and well-being of children in foster care and their caregivers (in Texas, USA), pointing out disparities within the CWS system and the unique stressors experienced by minority children due to barriers to accessing health care services, and identifying effective strategies (including telehealth) in addressing these barriers to care. Four focus groups were held with 22 participants, while 14 responded via e-mail (pediatricians, behavioral health team, primary care providers in a clinic, and child welfare and health advocates) [31].

Greiner et al. [29] assessed the impact of telehealth on health care delivery in a children’s hospital serving youth in foster care in the USA. The study was motivated by the possibility to increase access for youth in foster care, eliminating barriers as transportation challenges and caregivers missing work. Data consists of health records among youth in foster care (*n* = 36 children and adolescents), clinical notes and a satisfaction survey via phone among users. Another study using health records, also in the USA (LA), was performed by Perez et al. [35] investigating telemental health (TMH) services. The aim was to utilize data to explore how client engagement among foster and adopted youth changed during the transition from in-person to TMH services. Furthermore, they explored the impact of client demographics, diagnosis, and treatment modality on engagement. The study was motivated by what was considered as “early evidence suggesting that TMH and other telehealth services are a practical and beneficial treatment approach for increasing engagement among youth” [35].

Leo et al. [30] aimed to understand CWS professionals’ (e.g. social workers, residential treatment staff, and supervisors) perspectives on enabling factors and barriers to providing family-based interventions via telehealth to youth in out-of-county foster care placement in the USA. They performed three semi structured focus groups with CWS professionals (*n* = 19) targeting (1) the current landscape of family-based interventions for foster youth, (2) perspectives on content topics for a family-based intervention, and (3) recommendations for telehealth delivery (eg, timing) [30].

### Results in studies under theme 1 (access to treatment):

Two of the three studies on teletherapy to trauma-experienced children, measuring treatment outcome of (one-to-one) videoconferencing, report a significant reduction of pre-post PTSD symptoms in their samples (*n* = 15 and *n* = 14) [33, 36]. Stewart et al. [36] conclude that their result is an important first step in determining how to best address the mental health needs of trauma-exposed youth with barriers to access to care. Martin et al.’s [33] study, however, showed low completion rate among young people in foster care (*n* = 14 of 46) explaining this as a consequence of placement change; new foster home or being admitted into a residential treatment center.

In Baker et al.’s [27] survey concerning the same target group (children who have experienced trauma), clinicians (*n* = 250) reported that teletherapy made logistical aspects of treatment easier and helpful especially with respect to scheduling, convenience, access and utilization of services. Specifically, they reported that families had an easier time scheduling and attending sessions because there was no cost or travel time involved in attending the therapy session [27].

Perez et al. [35] compared client engagement changes in the shift from in-person to telemental health services among foster and adopted youth. They found a higher number of sessions (via health records), briefer sessions and more time in therapy in total in telemental health. Moreover, they found an increased client engagement attributed to the ease and accessibility of telemental health. Greiner et al. [29] however, found reduction (via health records) in show-up rates for adolescents in foster care for telehealth visits versus in-person. Leo et al.’s [30] focus group study among CWS professionals (*n* = 19) showed their optimism about telehealth both in terms of improved access to consistent care, remove transportation barriers, and better match diverse or specialized providers to youth.

The study by Eapen et al. [28], based on a lexical analysis among clinicians (*n* = 6), reports that e-mental health can extend the reach of and access to psychiatry services, particularly for individuals disadvantaged by inequity. Two studies report on the use of telepsychiatry in improving access to care through specific programs to improve providers’ telepsychiatry skills [32, 34]. Malas et al. report from their survey among primary care providers (*n* = 649) that telepsychiatry access to specialist expertise improved patient care for youth with mental illness (45.3%), improved comfort and confidence in caring for youth with mental illness (30.9%), provided greater comfort with the prescribing and monitoring of psychotropics (25.9%) and improved access to mental health care (23.1%) [32]. The study by Mundt et al. shows that clinicians in residential care under CWS found that contact with specialists helped them to diagnose and treat children and improved treatment capacity [34].

In the study of Loria et al. [31], the health care provider participants (*n* = 36) considered telehealth to be an effective strategy for engaging patients in essential health care as well as enabling foster children to connect with parents across distances. The study emphasizes impacts that may have been particularly felt by families living in rural and underserved communities. Some health care providers reported decreased no-show rates and that being able to see children in their home environments provided insight into the child’s living situation, while children were more relaxed and open in some cases. Several disadvantages of video consultations are mentioned in the ten studies under theme 1. Several stressed technological issues; the problematic nature of dependence on technological skills, access to equipment and the internet, and problems with privacy that may arise (e.g., if the child or young person does not have access to a separate room) [28, 30, 31]. Video consultations can prevent clinicians from examining the home situation, monitoring development, and assessing risks and needs for CWS [28]. However, the opposite perspective is emphasized by clinicians (*n* = 7) in Martin et al.’s [33] study who discussed “how telehealth allowed for a better perspective on the patient-caregiver dynamic because treatment took place in a more natural setting (i.e., the child’s home)”. On the other hand, the clinicians also noted problems such as young people having screen fatigue, shorter attention span and more distractions. The clinicians experienced struggling with keeping them engaged—particularly problematic in the case of young people with trauma [33].

Other negative aspects mentioned are uncertainty about how to develop a therapeutic relationship, as well as ethical questions, the lack of an evidence base and strategies for the provision of trauma-informed care in the virtual setting [31]. In Baker et al. [27] many mental health clinicians reported that trauma work was not ideally suited for remote therapy. They also reported interpersonal aspects of mental health treatment as more challenging via telehealth, assessing the child’s feelings and supporting the child emotionally. Technology-related aspects were also challenging, especially for children, as well as attention span and screen fatigue [27].

The studies were mainly based on the experiences of health and social care workers when reporting the advantages and disadvantages of videoconferencing in treatment. Despite disadvantages that may greatly affect care quality, the studies also indicate several reasons why it is perceived as useful. There was broad agreement that it provides improved access to specialized health care and that it may overcome barriers related to distance, social inequality, and poverty. Two of the studies concluded that it was useful to have combined models of face-to-face and video sessions [28, 34].

The three studies on teletherapy delivered to traumatized youth indicated a need for more knowledge about patient satisfaction and cost-effectiveness in video-based treatment [36]. Additionally, to build upon and extent the findings [27] and more research to better understand the benefits and limitations of telehealth delivery of TF-CBT for young people in the child welfare system [33]. More generally, others were concerned with how videoconferencing affects practice, knowledge and results for patients and families in the longer term [32]. Further areas in need of exploration were the experiences of clinicians and patients, identification of factors that affect this form of treatment and testing different models to optimize it [28], research on clinical effects in vulnerable populations with scaling to several clinics and with a larger patient population [34], and research on trauma care [31]. More research to ensure that increased no-show rates is avoided is suggested [29].

## Theme 2: young people’s perspectives

Four studies explore the experience of adolescents and young people in care and care-experienced on use of videoconference in mental health treatment [41–44].While two of the studies included adolescents and young people only [41, 43], two studies also including providers and carers as informants [42, 44]. The four studies were published in the period 2022-2024.

Three of the studies were qualitative [41, 42, 44], and one used mixed methods combining survey and interviews [43].

### Objectives and methods in studies under theme 2 (young people’s perspectives):

Krane et al. [43] study how young people receiving child welfare services experience the use of video consultation in mental health treatment in Norway, and how they experience the therapeutic relationship. The study has a mixed methods design including qualitative interviews (*n* = 10 aged 15–19) and a quantitative survey with young people receiving child welfare services. The survey included 232 participants aged 16–24, of which 36 reported having received video consultations in mental healthcare [43].

Archard, Kulik et al. [41] did an evaluation of a child and adolescent mental health team based in the NHS in England. The aim was to explore changes in practices during the pandemic and understand experiences of and satisfaction young people under the care of this service: a) the care and treatment received and b) communication with the team at this time [41]. The study is based on interviews with users; children and young people (*n* = 16) living in residential and foster care, adopted, and involved with youth justice services.

Stabler et al. [44] explored stakeholders experience of online mental health interventions and services for children in care and care-experienced young people, their need to protect their freedom to take risks and access digital spaces like other children while also recognising the pressures on practitioners and carers to protect them from harm. The study involved online one-to-one and small group interviews with young people with experience of care (*n* = 3); a young person whose biological parents were foster carers (*n* = 1); foster and kinship carers (*n* = 10); and social care and affiliated professionals (*n* = 9). Evans et al. [42] build upon the same data material as in Stabler et al. [44], exploring experiences of delivering, supporting or receiving mental health and wellbeing interventions online or remotely during the COVID-19 pandemic. The study’s findings were refined and confirmed through a stakeholder consultation group.

### Results in studies under theme 2 (young people’s perspectives):

The results in Krane et al. [43] show that young people in contact with the child welfare service experienced video consultations as more superficial and less binding compared to in-person sessions within CAMH-services. Participants raised concerns about the therapeutic relationship, with many finding it much more difficult to communicate effectively on screen, and several reported that their relationship with the therapist worsened. Additionally, a significant proportion (42%) claimed that video consultations did not meet their treatment needs. However, a minority of participants found it easier to regulate closeness and distance and preferred talking to the therapist on screen [43]. Archard, Kulik et al. [41] studied 16 young people in care provided by a specialist CAMH team and found overall satisfaction with the service. The study highlighted that therapeutic relationships with clinicians held renewed significance when care was delivered remotely or through a combination of remote and face-to-face interactions. Stabler et al.’s [44] explored different stakeholders' experiences, including four young people in foster care, reflecting on the complexities of online communication. The study pointed out that living arrangements can restrict access to services and complicate confidentiality, making young people and their carers reluctant to discuss personal matters [44]. Similarly, Evans et al. [42] emphasized the need for safe and private spaces for young people accessing online services and noted a lack of knowledge regarding online support for care-experienced young people, who require both choice and flexibility in their treatment options.

All four studies highlight the need for more research based on adolescents and young people’s views. Based on the advice from young people more research is suggested on how they can most efficiently access and gain awareness of mental health and wellbeing support as well as more in-depth exploration of online safety [42]. Furthermore, research which recognises the specific needs and environment of young people in care as well as their carers is required [44]. Moreover there is a need for research including young people’s views to ensure that care pathway planning is oriented to their needs [41] and to examine how user involvement can be incorporated into VC therapy and how this could improve experiences and quality of VC [43].

## Theme 3: videoconferencing in interdisciplinary meetings

The main theme in three of the articles was videoconferencing in interdisciplinary meetings between CAMHS and CWS and other services [26, 45, 46]. The studies were conducted in 2021–2022, and all focused on the impact of COVID-19 on service provision and availability, and on maintaining contact, continuity, and quality of care. Two studies were quantitative [45, 46], and one qualitative [26].

### Objectives and methods in studies under theme 3 (VC in interdisciplinary meetings):

Archard, Fitzpatrick et al. [45] explored consultation practice in an interdisciplinary team for mental health services for children and young people before and during COVID-19, based on administrative data from 258 consultations.

Baginsky and Manthorpe [26] examined how the disruption caused by COVID-19 led to new ways of communicating, how it affected the professions, and the potential long-term impact. Their qualitative study contained case studies with 40 interviews with 46 representatives from various services in England, and some informants from services in five other countries in Europe [26].

Coon et al. [46] evaluated the extent to which service indicators for a state-funded team working with foster youth changed after the service delivery model changed from in-person to telehealth services. The study was based on a descriptive case study using administrative data, and a brief survey of practitioners’ satisfaction with the transition to telehealth services.

### Results in studies under theme 3 (VC in interdisciplinary meetings):

The studies underlined the practical advantages of videoconferences in terms of saving traveling time. The advantage of reduced travel costs was pointed out [46], but also of increased attendance by the service providers involved because it was easier to participate via video [26]. Coon et al. [46] also reported that a team that worked with foster families received a significant increase in appointments, and better continuity in cases involving behavioral interventions. On the other hand, the numbers of client contacts, intake and closing of cases did not significantly decrease. Baginsky and Manthorpe [26] reported that some of the informants found that collaboration improved with digital meetings, and that service providers were eager to learn from each other.

It was also reported that virtual collaboration meetings had some potential negative aspects, influencing the quality of the contact in meetings. With most communication and meetings online in the team, both Archard, Fitzpatrick et al. [45] and Baginsky et al. [26] expressed concern for families who are digitally disadvantaged. Archard, Fitzpatrick et al. [45] were concerned about the need for a greater focus on the most vulnerable groups, as the landscape of mental health and service delivery changed during COVID-19. In their study on specialist mental health teams aimed at social workers, they found a tendency for social workers to attend virtual meetings with the camera turned off, thus making it difficult to pick up subtle signals in body language and “emotional atmosphere”.

According to the included studies, digital collaborative meetings can have important advantages over physical meetings, such as increasing attendance and meeting frequency. In turn, this can provide better continuity of care as well as familiarity with and understanding of each other’s fields of expertise. Some services may acquire a greater role and impact. Baginsky et al. [26] suggest that attention should be paid to people who were digitally disadvantaged (i.e., lacked the necessary technology or skills to participate in virtual meetings).

The three studies on videoconferencing in interdisciplinary collaboration emphasized the need for more research on the effects this will have on children and their families, and in different places [26], and the need to explore different learning opportunities where teams work (digitally) with caregivers, such as communication techniques and social skills training [46].

## Theme 4: use, awareness, and acceptance of videoconferencing in care services

Five of the articles explored the use, awareness, and acceptance of videoconferencing in health and social care services [47–51]. One was quantitative [50] and the rest qualitative [47–49, 51]. One study was from before COVID-19 [47], while the remaining studies relate to the pandemic, studying changes in CAMHS and CWS services caused by a situation with COVID-19 restrictions in care services, which made the use of videoconferencing increase in a very short time. An important discussion in this research is whether care services will continue to use videoconferencing in their future work.

### Objectives and methods in studies under theme 4 (use, awareness and acceptance of VC):

Mishna et al. [48, 49] conducted two studies on providers’ use of technology before and after COVID-19 based on interviews with a sample of 27 practitioners and 22 clients in a large urban centre in Ontario, Canada. Eleven of the practitioners were interviewed both prior to and during COVID-19 [48, 49]. The study by Pink et al. of video calls and other digital practices included data from interviews with 29 social workers, ten social work managers, and nine family support workers in England [51]. The study by Molfenter et al. [50] on the adoption of technologies, acceptance of these technologies and intentions of providers to use telehealth was based on a sample of 327 mental health organizations from 22 US states during May–August 2020. The respondents were clinicians and administrators in health and social services.

The only study from before COVID-19, Mackrill and Ebsen’s [47] qualitative study, reviewed misconceptions regarding the assessment of digital technology for youth social work in municipal contexts via data from field notes from an innovation (app development), where attitudes towards digital technology were studied.

### Results in studies under theme 4 (use, awareness and acceptance of VC):

Mishna et al. [49], in their study among social workers before and during the pandemic, characterized developments during COVID-19 as a paradigm shift in the use of Information and communication technology in social work. They underlined that this transition had increased awareness of the availability of services and awareness of confidentiality and personal protection, since COVID-19 brought new issues to be dealt with. In the second study by Mishna et al. [48], the authors describe increased access for some users, but also several barriers to telecare: video calls were distracting, a lack of internet access and poor digital literacy [48].

Molfenter et al. [50] in their study of health and social workers in 22 states in the USA from May to August 2020 showed widespread use of technology and found that people were positive about video-based services, that the majority intended to use technology after COVID-19, and that video was preferable to telephone calls [50]. Mackrill and Ebsen [47] and Pink et al. [51] both studied the relationship between social work and digital technology, in Denmark and England, respectively. Mackrill and Ebsen [47] found that service providers were often expected to use digital technology without training, and pointed out a number of misunderstandings in the assessment of digital technology. They argued that the relationship between social work and digital technology is complex, and that this complexity must be understood in developing digital technologies. Pink et al. [51] found that digital social work could offer something different rather than something worse, and argued for the concept of “digital social work” as a hybrid practice, which would be inevitable in future social work.

The studies show a paradigm shift in services, based on the introduction of technology for communication. In most cases the development was accepted and desired, and led to increased awareness in social work. There are many indications that this is an inevitable development, and that the challenges ahead, according to Mishna et al., will be to use the opportunity to develop client- centered models of service delivery.

Mishna et al. argued that research is needed on the implications of these technological changes for both clients and social workers, including the use of videoconferencing. Molfenter et al. [50] propose research on the effects of virtual services, on outcomes for patients, and on the degree of acceptance of the technologies. They believe that practice and research are particularly important to find ways to integrate in-person, telephone and video-based services to achieve a high degree of patient-centeredness and the best possible results [50].

## Discussion

This scoping review explored current knowledge on the use of videoconferencing in CAMHS for children receiving CWS and in collaborative meetings between CAMHS and CWS, based on findings from 22 peer-reviewed empirical research articles published between January 2012 and April 2024. The key findings of this review raise questions related to videoconferencing in terms of the following main themes: access to specialized mental health treatment, quality of care and treatment, and effectiveness of collaboration between CAMHS and CWS. After presenting the range of the literature, we will now discuss these aspects and indicate research gaps and implications for practice.

### Range of the literature

There were relatively few studies 22 that met the inclusion criteria in this scoping review. While there are many studies and several reviews of videoconferencing in CAMHS in general, an important reason for the relatively low number of studies in this review is that our study population was limited to children and young people in CWS or other specific related vulnerable groups (without CWS being particularly mentioned). Most of the studies were published in 2021–2024, investigating videoconferencing during or after COVID-19.

An important limitation to the range of the literature is that while many of the studies included service providers’ responses (*n* = 17) few studies included data based on responses from children, adolescents or young people (*n* = 5). The twelve qualitative studies included individual interviews, focus groups, case studies, lexical analyses, and digital observations. The eight quantitative studies mostly included surveys among providers or health records and descriptive administrative data on consultation practices and more general data on client contacts, appointments, intakes etc. Two studies combined qualitative and quantitative methods. No randomized controlled trials were found in the search. Hence, the research identified was relatively narrow as the studies did not include clients’ own experiences and perspectives, mental health outcomes, or effect studies of videoconferencing or interdisciplinary meetings.

### Videoconferencing to increase or ensure access to treatment

Access to specialized mental health treatment for children and adolescents receiving CWS, and related underserved, vulnerable groups with various barriers to treatment, forms the background to most studies in this scoping review. Moreover, many of the studies were conducted during COVID-19 and after, aiming to explore videoconferencing as an alternative way of reaching children and their families or ensuring continuity of care, and the lessons learned from this by providers. COVID-19 immediately led to increased attention to questions of access to services, primarily how to maintain contact with vulnerable groups of children and families who already had various barriers to treatment [52].

This scoping review shows that the literature on videoconferencing in treatment and in collaboration between CAMHS and CWS aims to reduce barriers to treatment and explore video-based solutions for mental health care to improve and ensure access or to maintain contact and continuity of care [26, 28, 30, 31, 34, 36, 41, 45, 46]. The studies indicate that videoconferencing, either directly through one-to-one treatment or indirectly through health and social workers’ access to and advice from specialists, improved access to mental health treatment for children and adolescents receiving CWS or other related vulnerable groups with specific barriers to treatment [27, 28, 31, 32, 34, 36, 41, 45]. In addition to improved access to specialized mental health care by overcoming barriers such as distance, social inequality and poverty, practical benefits are mentioned, such as saving traveling time for patients, families, and therapists. This could lead to greater efficiency, less no-show, increased patient flow and reduced waiting lists for treatment in CAMHS. While the studies mention and discuss such outcomes based on health and social care providers’ observations and impressions, none of them systematically measured the effect of videoconferencing on accessibility.

In terms of access to treatment, some of the studies also emphasize negative factors, such as poorer treatment access due to dependence on and access to equipment and competence to use it [28, 48]. Two studies showed low completion rate and show-up rates – both among adolescents and young people in foster care [29, 33]. Other studies warn that at worst the use of videoconferences can exacerbate disparity due to structural inequalities in access to digital tools [53, 54]. Videoconferencing also raises new privacy issues, where access to a private space may not be possible for some of these young people [42, 44] the young people’s living arrangements can restrict access to services and complicate confidentiality [44]. This is an important limitation mentioned as a general pitfall in e-health [23].

### Quality of treatment

Except for Stewart et al. [36] and Martin et al. [33], none of the other studies measure outcomes of treatment via exploring videoconferencing in a pre-post-design, and only a handful of the reviewed studies explored children, adolescents or young people’s own perspectives on the quality of treatment. Two of these included other stakeholders as well, and only very few included young people [42, 44]. Aspects of particular concern was establishing a good therapeutic relationship and young people’s ability to be focused in virtual treatment [31, 41, 43]. While Archard, Kulik et al. [41] concluded that young people’s (in residential- or foster care etc.) therapeutic relationships with clinicians appeared to hold a “renewed significance”, Krane et al.’s [43] study revealed important weaknesses and disadvantages of online therapy as experienced by young people in child welfare services (including residential- and foster care). Krane et al. [43] described their result as particularly worrying with reference to the relational aspects of treatment, as children receiving CWS often have relational experiences which make them particularly sensitive to challenges in relationships. This perspective is in line with other studies showing that the relational experience of treatment deteriorated in videoconferencing in CAMHS [23, 55]. In general, a good therapeutic relationship is considered the key element in psychological treatment [56–58]. Moreover, children in CWS have often experienced difficult and turbulent relationships, which can make them particularly sensitive to relational challenges [18]. Such challenges should be carefully assessed when considering video-based treatment. Another concern is that it is not possible to monitor development or to perform risk assessment in the same way as in face-to-face meetings [28, 41], which is a particular concern for digitally disadvantaged families [26, 49]. This might pose a specific risk to children and adolescents in vulnerable care situations. Concerns and questions about safeguarding and confidentiality for vulnerable groups in videoconferences have also been raised in other studies [59–61].

None of the studies included user participation in the sense of letting the users provide feedback of the digital service to take account their perspectives, opinions and input at an individual level in therapy nor at a structural level designing the services. However, Stabler et al.’s [44] study included consultations with young people both to reflect on the study and indicate important implications for future research. The lack of studies involving user participation to inform future service design has also been mentioned elsewhere [62].

Based on the findings of this review, an important question is whether videoconferences have a quality and format that covers the needs of children and adolescents receiving child welfare services.

### Effective interdisciplinary collaboration

The reviewed studies showed clear benefits of using videoconferences in interdisciplinary meetings between CAMHS and CWS, as well as in consultation sessions with specialists [26, 45, 46]. The studies showed that collaborative videoconferences increased attendance at and frequency of important meetings between social services and mental health services. They also suggested that this led to better knowledge of each other’s perspectives and continuity in interdisciplinary collaboration as well as familiarity with and understanding of each other’s fields of expertise. On the negative side, it was also pointed out that it is important to consider what kinds of topics are suitable for discussion, and what topics that should be avoided, in videoconferences. A further point is whether videoconferences will lead to reduced participation in collaborative meetings by families and children, i.e., reduced service user involvement. None of the reviewed studies explored young people’s, parents’ or caregivers’ experiences and perspectives on the use of videoconferencing in collaborative meetings. The three studies exploring interdisciplinary work were all conducted during COVID-19. Key questions are whether telehealth practices will shape future work after COVID-19 and whether they enhance understanding [26].

### A paradigm shift taking place?

Several of the included studies described in different ways that a paradigm shift is taking place based on a large increase in technology use, including videoconferencing [47–51]. The main impression from the included literature is that this development is accepted and desired by service providers and health authorities, and that it has led to increased awareness of some aspects of the work. Some define it as an inevitable development and that the challenges going forward will be to use the opportunity to develop good client-centered models for service delivery [47].

The concerns about technological developments mentioned in the literature are important to consider. This applies particularly to the quality of the therapeutic alliance in video-based treatment [31, 42]. The lack of quality assessment raises concerns about videoconferencing in CAMHS for children receiving CWS. A naïve non-evidence-based use of videoconferencing aimed at this group or other groups with similar vulnerability can at worst create new barriers to CAMHS treatment. There is still a need for research that examines in which cases and for whom it is an advantage, if the goal is to increase access to and quality of treatment [63]*.* Although videoconferencing may improve access to treatment, it is not beneficial if young clients find that treatment quality and effect deteriorate because it is harder to talk to the therapist and establish a good relationship.

### Research gaps

If videoconferencing is to become part of regular treatment in CAMHS directed at children and adolescents receiving CWS or similar vulnerable groups, more research is needed. This includes research on clinical outcomes as well as consequences of videoconferencing in collaboration between CAMHS and CWS. The literature reviewed emphasizes the need for more knowledge about providers’ and patients’ experiences and satisfaction with videoconferencing in treatment and in interdisciplinary collaboration. There is also a need for insight into how it affects practice, knowledge and results for patients and families in the longer term. The need for more research on clinical effects is particularly emphasized. In terms of videoconferencing in collaboration between service providers and with caregivers, the research indicates a need to explore communication techniques and social skills training [46].

The reviewed studies are mainly based on the experiences of health and social care providers. Further studies should include children, adolescents and families receiving CWS and explore their experiences and perspectives on videoconferences in CAMHS treatment. Moreover, none of the studies included service user involvement in decisions and development of videoconferencing in CAMHS directed at children and adolescents in CWS. In further development of videoconferencing for the CWS population, a service user perspective must be included in the design of practice and in research projects. Involving people with lived experience, such as young people and/or their parents, in future research as part of project working groups is crucial to ensure the research addresses and meets the specific needs of this group.

### Implications for practice and policy

Although videoconferencing has been used for over twenty years in mental health services for children and young people in many countries, and despite the huge increase in its use during COVID-19, there are still questions about confidentiality, treatment quality and its effects on health and inequality [4, 62, 64]. In particular, there are unanswered questions concerning vulnerable groups with complex health and social care needs, whether videoconferencing reduces or exacerbates inequalities in access to mental health care, and whether it strengthens or weakens rights to involvement and participation [62]. Furthermore, the importance of clinical training and skill development should not be underestimated.

In line with this review, policymakers and practitioners in this field should consider these important issues in the further use and development of videoconferencing. This is especially important as young people receiving CWS are in a vulnerable care situation. It must be realized that there is insufficient evidence to determine whether videoconferencing offers high-quality treatment to this target group. Practitioners should be especially aware of the fact that therapeutic relationships can be difficult to develop online, and that this target group already has many barriers to mental health treatment and will be particularly vulnerable to new therapeutic obstacles. It is also vital to implement strategies that strengthen service user involvement and participation in the further development of videoconferencing in mental health care for young people receiving CWS.

### Strengths and limitations

A general limitation of scoping reviews is the lack of critical quality assessment of the included studies [24]. However, the articles included in our scoping review were all peer-reviewed, which may mitigate some concerns about quality. Compared to a systematic review, a scoping review will include literature with a wider range of study designs and methodological approaches [65]. The way we defined our topic influenced our search terms, potentially limiting the breadth of the studies included in our review and thus affecting the generalizability of our findings. In contrast to systematic reviews that examine “what works” questions, scoping reviews allow for a wider range of methods and usually do not include an assessment of the quality of the methods used in specific studies [66]. As described in Peters et al. [67], scoping reviews can be particularly useful to explore literature in different disciplines, and are suited to address questions beyond effectiveness or experience of an intervention. While some scoping reviews mainly focus on quantifiable measures (e.g., year of publication, type of study design, location of research, etc.), a strength of our scoping review was that we performed and described a detailed thematic analysis of the articles included.

We may have missed some relevant studies due to the choice of databases searched. Furthermore, we did not include other languages than English, and we excluded books and gray literature. In terms of concepts describing child welfare services, we went through several other reviews to ensure that our search did not overlook essential terms denoting the service. The main purpose of child welfare services, as defined by legislation, is relatively similar across countries, even if the systems for protecting children differ.

The generalizability of the findings can be questioned due to the predominance of qualitative studies and the low number of participants in the quantitative studies.

## Conclusion

This scoping review shows that from a service provider perspective, videoconferencing increases access to CAMHS treatment for children and adolescents receiving help from CWS and improves interdisciplinary collaboration between CAMHS and CWS. However, research is lacking on clinical outcomes and the service user perspective of videoconferencing. If videoconferencing in mental health services is to become an established and trusted method aimed at children and adolescents receiving CWS, several unresolved and potentially negative issues need attention and more research. This is especially relevant concerning the inclusion of children's and young people’s perspectives in research on VC therapy and in the design and development of digital services throughout the research process. Moreover, whether videoconferencing reduces or exacerbates inequalities in access to mental health care, and whether this group of children and adolescents are less likely to seek help from a therapist in a digital rather than an in-person face-to-face setting. A further question is whether new barriers are raised by screen-based treatment to threaten good therapeutic relationships, and by extension treatment quality and clinical outcomes.

### Supplementary Information

Supplementary Material 1.

## Data Availability

Datasets used and analyzed in this scoping review are available from the corresponding author on request.
