# Supplementary material for: Videoconferencing in mental health services for children and adolescents receiving child welfare services: a scoping review
Source: BMC Health Serv Res. 2024 Jun 14;24:729. doi: 10.1186/s12913-024-11157-y (PMC11177372; doi:10.1186/s12913-024-11157-y)
Supplement: Supplementary file 1 — Supplementary Material 1. [file 12913_2024_11157_MOESM1_ESM.docx]

**Table S-1. Overview of 22 articles included in the scoping review**

| Main theme | Articles | Service setting and methods | Results videoconferencing (VC) /telehealth | Conclusion |
| --- | --- | --- | --- | --- |
| VC in treatment | Stewart et al. (2017). A Pilot Study of Trauma-Focused Cognitive-Behavioral Therapy Delivered via Telehealth Technology. *Child Maltreat, 22(4), 324-333.* | Pilot study of trauma-focused cognitive– behavioral therapy (TF-CBT) delivered to underserved trauma-exposed youth (n=15) via VC, aimed at addressing barriers in access to treatment. | - Significant effect on PTSD symptoms - Important first step regarding the health needs of particularly vulnerable groups - Minimal technical difficulties - Better access | Telehealth TF-CBT had promising results in terms of treatment effects. One-one videoconferencing can be compared with a physical setting. |
| VC in treatment | Martin et al. (2023) Examining the Feasibility of Telehealth Trauma-Focused Cognitive Behavioural Therapy (TF-CBT) with Young People in Foster Care. *J Child Adolesc Trauma, 16(4), 1-9*. | Telehealth trauma-focused cognitive– behavioral therapy (TF-CBT) delivered to young people in foster care in the US. Health records (n=46) and focus group study with mental health providers (n=7). | - Significant effect on trauma symptoms - Low completion rate (n=14) - Barriers to treatment completion - Issues in home environment, caregiver participation, and systemic topics | Telehealth TF-CBT with young people in foster care is feasible but there is a potential need for greater emphasis on coordination and continuity of mental health treatment for youth in the child welfare system. |
| VC in treatment  -providers’ experience | Baker et al. (2023) Successes, challenges, and opportunities in providing evidence-based teletherapy to children who have experienced trauma as a response to Covid-19: A national survey of clinicians. *Children and Youth Services Review, 146, 1-10.* | Teletherapy delivered to traumatized children (as a response to Covid-19) across the USA. Survey among clinicians (n=250). | - Logistical aspects of treatment easier - Some clinical aspects of care easier engagement with caregivers) - More challenging to keep children’s attention and to assess emotions | The results suggest many avenues for refining and fine-tuning remote mental health services, especially for children. |
| VC in treatment -providers' experiences | Malas et al. (2019). Exploring the Telepsychiatry Experience: Primary Care Provider Perception of the Michigan Child Collaborative Care (MC3) Program. *Psychosomatics, 60(2), 179-189.* | Telepsychiatry service program (including video-consultation), with integrated behavioral and health consultations in primary care in the USA. Survey among primary care providers (n=649). | - Better access to mental health care - Better quality of help - Increased confidence in connection with medicines | The program was well accepted in the primary health services, and the service providers consider that they provide a better service through this program. |
| VC in treatment -providers' experiences | Eapen et al. (2021). E-mental health in child psychiatry during COVID-19: an initial attitudinal study. *Australasian Psychiatry, 29(5), 498-503.* | Child and Adolescent Psychiatric Service in Australia. The study includes graduates and their supervisors, (n=5+1) reflecting on advantages and disadvantages of e-mental health, including videoconferencing. | - Better access to mental health care - Dependent on technological competence, equipment, internet access, physical space/privacy - Can hinder clinicians' ability to examine, monitor development, assess risks and the need for child protection | A combined model with both face-to-face and VC can be valuable. It will require practical, flexible guidelines that protect children's and families' privacy and respect the needs and preferences of children, families, and clinicians. |
| VC in treatment -providers' experiences | Mundt et al. (2021). Telepsychiatry consultation for primary care treatment of children and adolescents receiving child protective services in Chile: Mixed methods feasibility study. JMIR Public Health and Surveillance, 7(7*), 1-7.* | Children and adolescents living under the supervision of child protective services in rural Chile treated in a telepsychiatric consultation program. The study includes 15 sessions with 11 children and 8 clinicians. | - Implementation of VC perceived as useful - Supported the clinicians in diagnosing and treating children in child welfare services with psychosocial needs - Improved treatment capacity - Technical problems solved in most cases | VC was perceived as useful as a support for clinicians in the follow-up of institutionalized children and young people in areas with few resources. Research is needed on the clinical effects in this population. |
| VC in treatment -providers' experiences | Loria et al. (2021). Multiagency working between children’s social care and schools during COVID-19: case study experiences from English local authorities and international reflections. *Journal of public child welfare, 17(1), 1-24.* | Health- and welfare providers working with children in foster care in Texas, USA, using telehealth with children in foster and kinship care. The study includes four focus groups held with providers in healthcare, mental health, and child welfare. | - Insight into home situation - Decreased "no-show" (better access) - Uncertainty about how to develop a therapeutic relationship - Lack of equipment among young people - Inability to stay focus when on screen - Lack of technology knowledge | Need to increase access to services for children in child protection measures, ensure that standards of care are met, and create innovative solutions to build capacity. |
| VC in treatment  –providers' experiences | Leo et al. (2023) Implementation Considerations for Family-Based Telehealth Interventions for Youth in Foster Care: Focus Group Study With Child Welfare System Professionals. *JMIR Form Res, 7:e45905* | Telehealth as a tool for mitigating barriers to access to treatment interventions for foster youth and their families in the USA. Semistructured focus groups (n=3) with child welfare system professionals (n=19). | - Optimism about better access to and more consistent mental health care - Some concerns regarding telehealth access and literacy | Highlight the need to develop policies and telehealth interventions that are youth versus placement centered, include resources that limit barriers and bolster motivation for engagement and follow a team-based model. |
| VC in treatment  -health records | Greiner et al. (2023) The Use of Telehealth for Youth in Foster Care. *Clin Pediatr (Phila), 62(12), 1465-1469.* | A children’s hospital serving youth in foster care in the USA. Assessing the impact of telehealth on health care delivery through health records (n=36 children and adolescents), clinical notes and a simple satisfaction survey among users via phone. | - Telehealth comparable to in-person visits in terms of history obtained - Reduction in show rates for adolescent for telehealth visits versus in-person - In-person visits resulted in more diagnoses and treatment recommendation | Telehealth was a generally acceptable platform for history gathering for youth in foster care. |
| VC in treatment  -health records | Perez et al. (2023). Mental health engagement among foster and adopted youth: the transition from in-person to telemental health services. *Social Work in Mental Health, 21(2), 203-221.* | Telemental health (TMH) services among foster and adopted youth explored via Health records (n=55). | - Higher number of sessions in telemental health - Briefer sessions than in-person sessions - More time in therapy in general - Increased engagement attributed to the ease and accessibility of telemental health | Telemental health can be used as a method to engage youth with foster care experience and their caregivers, which is critical given their need for mental health services. |
| VC in treatment  -young people’s experiences | Archard et al. (2022) Young people’s views on specialist mental healthcare and remote delivery during the COVID-19 pandemic. *Mental Health Practice.* Doi. 10.7748/mhp.2022.e1596 | Specialist child and adolescent mental health service team (CAMHS) in England, serving vulnerable groups. Interviews with users; children and young people (n=16) living in residential and foster care, adopted, and involved with youth justice services. | - High level of satisfaction with the service overall - Therapeutic relationships with clinicians appeared to hold a renewed significance | Concludes by considering the implications of the findings for practice and care pathway planning and commenting on the value of service evaluations for illuminating issues that transcend local care. |
| VC in treatment  -young people’s experiences | Krane et al. (2023) “It's not like real therapy”: young people receiving child welfare services' experiences of video consultations in mental healthcare in Norway: a mixed methods approach. *BMC Health Services Research, 23 (1)* | Video consultation in specialist mental health treatment (CAMH) among young people receiving child welfare services. Survey with young people (n=36) and interviews (n=10). | - VC experienced as more superficial and less binding compared to in-person sessions - Easier to regulate closeness and distance - Concerns about the therapeutic relationship | There are important weaknesses and disadvantages of VC as experienced by young people receiving child welfare services and worrying that this involves the relational aspects of treatment. |
| VC in treatment  -young people’s experiences | Stabler et al. (2023) “I probably wouldn't want to talk about anything too personal”: A qualitative exploration of how issues of privacy, confidentiality and surveillance in the home impact on access and engagement with online services and spaces for care-experienced young people. Adoption & Fostering, *47(3), 277-294.* | Online mental health and wellbeing interventions and services for care-experienced young people in Wales, UK. Interviews with young people in foster homes (n=4), carers (n=8), kinship carers (n=2) and social care professionals (n=9). | - The physical space of the fostering home affects how children and young people access digital spaces - Tensions in the home environment limits privacy, confidentiality, and autonomy | The large-scale shift to online delivery risks excluding vulnerable groups of young people. |
| VC in treatment  -young people’s experiences | Evans et al. (2024) Acceptability, feasibility and perceived effectiveness of online and remote mental health and wellbeing interventions during the COVID-19 pandemic: A qualitative study with care-experienced young people, carers and professionals. *Children and Youth Services Review, 156, 1-8.* | Stakeholders’ experiences of delivering, supporting, or receiving mental health and wellbeing interventions online or remotely during the Covid-19 pandemic in the UK. Online interviews with experienced young persons: n=3, foster and kinship carers: n=10, social care and affiliated professionals: n=9. | - Mixed experience of online interventions - Lack of knowledge regarding online support to care-experienced young people - Young people need choice and flexibility - Young people need safe and private spaces when accessing online services, access to online support and technological devices - Carers need training on how to foster relationships online and ensure safety and child protection | The study findings offer insight into how interventions and services may be developed and optimised moving forward to ensure that they are meeting the needs of young people in care and maximize likely effectiveness. |
| VC in interdisciplinary meetings | Archard et al. (2021). Consultation in a Specialist Mental Health Team for Vulnerable Children before and during the Early Stages of the COVID-19 Pandemic: Audit Findings and Practice-Based Reflections. *Practice, 1-15.* | Team for the mental health of children and young people where child protection has taken over care, aimed at social workers and carers (England). Study based on administrative data from 258 consultations | - Tendency for social workers to participate in VC with the camera switched off - Difficulty picking up subtle signals in body language and emotional atmosphere | The pandemic is changing the landscape of mental health and service delivery. Important to reflect on function and practice. |
| VC in interdisciplinary meetings | Baginsky et al. (2021). Multiagency working between children's social care and schools during COVID-19: case study experiences from English local authorities and international reflections. *Journal of Integrated Care, 30(2), 134-145.* | Interdisciplinary collaboration between schools, social services, and health services in England. Interviews (n=40) with providers in social care, health services and schools (n=46). | - Increased attendance due to ease via VC - Eager to learn from each other - The school played a bigger role - Some perceived better collaboration - Concern for digitally disadvantaged families | The study identifies interdisciplinary work during the pandemic and factors that can shape the work further. The question is whether practice survives the pandemic, and whether a deeper understanding is achieved. |
| VC in interdisciplinary meetings | Coon et al. (2022). Eight Months of Telehealth for a State-Funded Project in Foster Care and Related Services: Progress Made and Lessons Learned. Behavior Analysis in Practice. *Behavior Analysis in Practice, 15, 1348-1360* | A state-funded team working with young people in foster care and adopted in the USA. Study based on survey among providers in the team about 25 cases, and a review of literature. | - The number of client contacts, intake, closing of cases were not significantly reduced - Agreements with families increased significantly and there was better continuity in behavioral measures - Reduced travel costs | Given these results, the team can continue to provide services via telehealth after the Covid-19 pandemic. It is important to consider the negative effects telehealth can cause. |
| Use, awareness and acceptance of VC in the services | Mackrill et al. (2018). Key misconceptions when assessing digital technology for municipal youth social work. *European Journal of Social Work, 21(6), 942-953.* | Social work in municipal preventive work (Denmark). Study based on field notes where attitudes towards digital technology were studied. | - There are several misunderstandings in the assessment of digital technology - Tendency for service providers to be expected to use digital technology without training | The relationship between social work and digital technology is complex, and the development of digital technologies must be based on an understanding of this complexity. |
| Use, awareness and acceptance of VC in the services | Mishna et al. (2021). Responding to COVID-19: New trends in social workers’ use of information and communication technology . *Community Ment Health Journal, 57(7), 1244-1251.* | Social work in four agencies in large cities (Canada). Study of providers’ use of technology before and after COVID-19, based on interviews with a sample of 27 practitioners and 22 clients. | - Increased access for certain users - Barriers to the service also created by ICT - Experienced VC as more distracting - Didn't get trained on physical appointments - Not everyone had access to a digital service | The Covid-19 context has provided a unique opportunity to reflect on the challenges of the service. Rather than trying to revert to the way things worked in the past, opportunity to develop client-centric models of service delivery. |
| Use, awareness and acceptance of VC in the services | Mishna et al. (2022). Social Work Practice During COVID-19: Client Needs and Boundary Challenges. *Global social welfare: research, policy & practice, 9(2), 113-120.* | Social work in four agencies in large cities (Canada). Study of providers’ use of technology before and after COVID-19, based on interviews with a sample of 27 practitioners and 22 clients. | - Paradigm shift in the use of ICT within social work - Effects of this transition (impact): - Increased awareness of accessibility - Confidentiality and privacy - Boundaries changed between work and private life | Research is needed on the implications of the changes in ICT use for users as well as for social workers – including the use of VC. |
| Use, awareness and acceptance of VC in the services | Molfenter et al. (2021). Use of Telehealth in Mental Health (MH) Services During and After COVID-19 . *Community Ment Health J 2021, 57(7), 1244-1251.* | Health and social services (counselors, social workers, psychologists, case managers and psychiatrists) (USA). Sample of 327 mental health organizations from 22 states. | - Widespread use of technology - The majority intended to use technology in the service after the pandemic - Use of video was more desirable than telephone | Health and social care staff positive about video-based services. Research is needed on how it affects the offer and effects for the patients. |
| Use, awareness and acceptance of VC in the services | Pink et al. (2022). Digital social work: Conceptualising a hybrid anticipatory practice. *Qualitative Social Work, 21(2), 413-430.* | Child protection in four local authority areas (England). Interviews with 29 social workers, ten social work managers, and nine family support workers. | - Blurred differences between video calls and physical meetings in the office - Digital social work can offer something different rather than something inferior | Argues for a concept of "digital social work" as a hybrid practice, understood as an inevitable and necessary element of social work practice. |
